# Supplementary material for: Understanding Low Vaccine Uptake in the Context of Public Health in High-Income Countries: A Scoping Review
Source: Vaccines (Basel). 2024 Mar 4;12(3):269. doi: 10.3390/vaccines12030269 (PMC10975033; doi:10.3390/vaccines12030269)
Supplement: Supplementary file 1 [file vaccines-12-00269-s001.zip › vaccines-2776155-supplementary.pptx]

## Slide 1
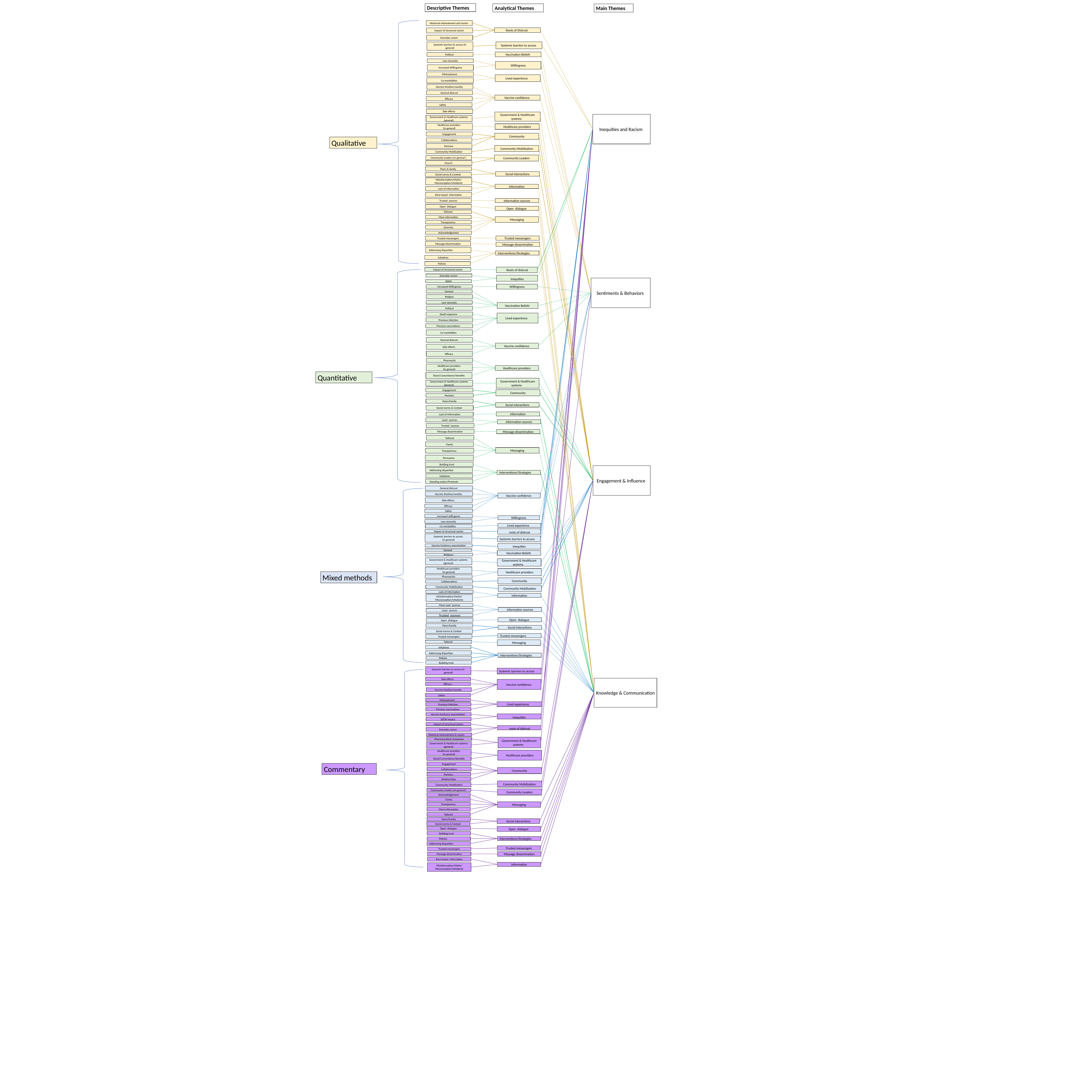

Descriptive Themes
Analytical Themes
Main Themes
Historical mistreatment and racism
Roots of Distrust
Impact of structural racism
Everyday racism
Systemic barriers to access
Systemic barriers to access (in general)
Vaccination Beliefs
Political
Low necessity
Willingness
Increased Willingness
Mistreatment
Lived experience
Co-morbidities
Vaccine timeline/novelty
General distrust
Vaccine confidence
Efficacy
Safety
Side effects
Government & Healthcare systems
Inequities and Racism
Government & Healthcare systems (general)
Healthcare providers
(in general)
Healthcare providers
Engagement
Community
Qualitative
Collaborations
Partners
Community Mobilization
Community Mobilization
Community Leaders
Community Leaders (in general )
Church
Peers & family
Social interactions
Social norms & Context
Misinformation/Myths/
Misconception/Infodemic
Information
Lack of information
Race-based information
Trusted sources
Information sources
Open dialogue
Open dialogue
Tailored
More information
Messaging
Transparency
Diversity
Acknowledgement
Trusted messengers
Trusted messengers
Message dissemination
Message dissemination
Addressing disparities
Interventions/Strategies
Initiatives
Policies
 Roots of distrust
Impact of structural racism
Everyday racism
 Inequities
Sentiments & Behaviors
SDOH
Willingness
Increased Willingness
General
Religion
Low necessity
Vaccination Beliefs
Political
Death exposure
Lived experience
Previous infection
Previous vaccinations
Co-morbidities
General distrust
Vaccine confidence
Side effects
Efficacy
Pharmacist
Healthcare providers
(in general)
Healthcare providers
Quantitative
Racial Concordance/benefits
Government & Healthcare systems
Government & Healthcare systems (general)
Engagement
Community
Partners
Peers/Family
Social interactions
Social norms & Context
Information
Lack of information
Least sources
Information sources
Trusted sources
Message dissemination
Message dissemination
Tailored
Clarity
Messaging
Transparency
Persuasive
Building trust
Engagement & Influence
Addressing disparities
Interventions/Strategies
Initiatives
Standing orders/Protocols
General distrust
Vaccine timeline/novelty
Vaccine confidence
Side effects
Efficacy
Safety
Increased willingness
Willingness
Low necessity
Lived experience
Co-morbidities
 roots of distrust
Impact of structural racism
Systemic barriers to access
(in general)
Systemic barriers to access
 Inequities
Vaccine hesitancy exacerbation
General
Vaccination Beliefs
Religious
Government & Healthcare systems (general)
Government & Healthcare systems
Healthcare providers
(in general)
Healthcare providers
Mixed methods
Pharmacists
Community
Collaborations
Community Mobilization
Community Mobilization
Lack of information
Information
Misinformation/Myths/
Misconception/Infodemic
Most used sources
Information sources
Least sources
Trusted sources
Open dialogue
Open dialogue
Peers/Family
Social interactions
Social norms & Context
Trusted messengers
Trusted messengers
Messaging
Tailored
Initiatives
Addressing disparities
Interventions/Strategies
Policies
Building trust
Systemic barriers to access (in general)
Systemic barriers to access
Side effects
Knowledge & Communication
Vaccine confidence
Efficacy
Vaccine timeline/novelty
Safety
Mistreatment
Lived experience
Previous Infection
Previous vaccinations
Vaccine hesitancy exacerbation
 Inequities
SDOH impact
Impact of structural racism
 roots of distrust
Everyday racism
Historical mistreatment & racism
Government & Healthcare systems
Pharmaceutical companies
Government & Healthcare systems (general)
Healthcare providers
(in general)
Healthcare providers
Racial Concordance/benefits
Engagement
Commentary
Collaborations
Community
Partners
Relationships
Community Mobilization
Community Mobilization
Community Leaders (in general )
Community Leaders
Acknowledgement
Clarity
Messaging
Transparency
More information
Tailored
Peers/Family
Social interactions
Social norms & Context
Open dialogue
Open dialogue
Building trust
Interventions/Strategies
Policies
Addressing disparities
Trusted messengers
Trusted messengers
Message dissemination
Message dissemination
Race-based information
Information
Misinformation/Myths/
Misconception/Infodemic
